# Treatment against coccidiosis in Norwegian lambs and potential risk factors for development of anticoccidial resistance—a questionnaire-based study

**DOI:** 10.1007/s00436-017-5400-7

**Published:** 2017-02-11

**Authors:** Ane Odden, Heidi L. Enemark, Lucy J. Robertson, Antonio Ruiz, Lisbeth Hektoen, Snorre Stuen

**Affiliations:** 1 Faculty of Veterinary Medicine, Department of Production Animal Clinical Sciences, Norwegian University of Life Sciences, Kyrkjevegen 332/334, 4325 Sandnes, Norway; 2 Norwegian Veterinary Institute, Ullevålsveien 68, P.O. Box 750 Sentrum, 0106 Oslo, Norway; 3 Faculty of Veterinary Medicine, Department of Food Safety and Infection Biology, Norwegian University of Life Sciences, P.O. Box 8146 Dep, 0033 Oslo, Norway; 4 Parasitology Unit, Department of Animal Pathology, Faculty of Veterinary Medicine, University of Las Palmas de Gran Canaria, 35416 Arucas Las Palmas, Spain; 5 Animalia Norwegian Meat and Poultry Research Centre, P.O. Box 396 Økern, 0513 Oslo, Norway; 6 Faculty of Veterinary Medicine, Department of Production Animal Clinical Sciences, Norwegian University of Life Sciences, P.O. Box 8146 Dep, 0033 Oslo, Norway

**Keywords:** Ovine coccidiosis, *Eimeria* spp., Anticoccidials, Norway, Drug resistance

## Abstract

The objectives of this study were to investigate the use of anticoccidials in Norwegian sheep flocks and identify farms with management procedures likely to select for drug resistance. Data were obtained by a questionnaire sent to all members of the Norwegian Sheep Recording System in October 2015. The data set consisted of 1215 answers, corresponding to 8.5% of Norwegian sheep flocks. Anticoccidials were used in 82.7% of flocks. Main treatment was at turnout (38.6% of treated flocks) or 1 week after turnout (32.4%). Interestingly, clinical signs possibly related to coccidiosis were observed by almost 40% of the farmers after treatment, which might be an indication of drug resistance. Correlations between the apparently reduced anticoccidial efficacy and management conditions, such as the size of the farms, were found. From the farmers’ perspective, metaphylactic treatment was used in 88.5% of treated flocks, of which approximately one third had no history of clinical coccidiosis. Even though farmers seem aware of the importance of good drenching routines based on reliable estimates of weights and calibration of drench guns, drench gun used for anticoccidial administration was never calibrated in 12.1% of the flocks. Finally, dose estimation was made by visual appraisal in 27.5% of the flocks, which can lead to incorrect dosing. Based on the present study, it cannot be determined whether the apparent treatment failure was related to management practises, incorrect administration of the drug, other infections or actual anticoccidial drug resistance.

**Electronic supplementary material:**

The online version of this article (doi:10.1007/s00436-017-5400-7) contains supplementary material, which is available to authorized users.

## Introduction

Coccidiosis caused by *Eimeria* spp. is a common cause of clinical disease and reduced growth in lambs (Chartier and Paraud 2012). Currently, 15 species are known to occur in sheep, of which 2 are considered major pathogens: *Eimeria ovinoidalis* and *Eimeria crandallis* (Rommel 2000; Catchpole et al. 1976; Catchpole and Gregory 1985). Depending on *Eimeria* species, the prepatent period varies from 2 to 3 weeks. The clinical signs include diarrhoea (occasionally haemorrhagic), abdominal pain, anorexia and weight loss/reduced weight gain (Wright and Coop 2007). Clinical disease is usually seen in young lambs with debut of symptoms 4 to 6 weeks post-partum depending on various factors, such as management and infection pressure (Gregory et al. 1980).

The lambing season in Norway is in March–May, dependent on geographical region. Lambs are weaned in the autumn, at around 4–5 months of age (Vatn 2009). During the summer, most ewes and lambs are moved to mountain or forest pastures, where the stocking densities are low: between 10 and 80 animals per square kilometre (Mysterud et al. 2001; Vatn 2009). Clinical coccidiosis is therefore primarily related to spring pastures with symptoms appearing 2 to 3 weeks after turnout (Helle 1964; Helle 1970).

Since ovine coccidiosis can have a major economic impact due to reduced weight gain and increased mortality, controlling the infection is important (Foreyt 1990; Alzieu et al. 1999). In 1987, Baycox® Sheep vet. (toltrazuril, Bayer Animal Health) was approved in Norway for treatment of coccidiosis as a single oral dose, and in 2007, Vecoxan® vet. (diclazuril, Elanco Animal Health) was marketed in Norway (Gjerde et al. 2009). Worldwide, several other drugs are licenced for treatment of ovine coccidiosis, e.g. decoquinate (Deccox®, Zoetis UK Limited). However, none of these other drugs are licenced for use in Norway (Norwegian Institute of Public Health 2015).

Anticoccidial resistance (ACR) in poultry has been reported against several anticoccidials, such as monensin, salinomycin, nicarbazin, halofuginone, robenidine, toltrazuril and diclazuril (McDougald 1981; Stephan et al. 1997). Testing for ACR in poultry can be done either by in vivo or in vitro assays (Chapman 1998; Thabet et al. 2015, 2017). However, despite the widespread use of anticoccidials in mammals, ACR has not yet been documented and no tests are available for livestock animals except for poultry.

Gjerde et al. (2009, 2010) reported reduced efficacy of Baycox® Sheep vet. in two farms on the southwest coast of Norway, thus prompting the need for more information on the use of anticoccidials in Norway. Additionally, several farmers have experienced an apparent lack of anticoccidial efficacy during recent years (Stuen S, personal communication). The aim of this study was to collect information concerning coccidiosis in lambs in Norway and the use of anticoccidials during the 2015 lambing and grazing season, with emphasis on identification of risk factors for anticoccidial resistance.

## Materials and methods

### Questionnaire

In October 2015, a questionnaire was sent by email to all members of the Norwegian Sheep Recording System (NSRS) with a registered email address using the Enalyzer Survey Solution (Enalyzer A/S). Of the 4781 farmers who were members in the NSRS, representing 33.5% of all sheep farmers in Norway, 3874 had a registered email address (Statistics Norway 2016a; National Sheep Recording System 2015). Farmers not responding to the questionnaire within 3 weeks were reminded once by email. In addition, the questionnaire was advertised in the Sheep and Goat Farmers’ Journal, a journal published six times a year, and subscribed to by 11,014 sheep and goat farmers (Norsk Sau og Geit 2015).

The questionnaire consisted of two sections: one concerning the general management of the flock, such as flock size, breed, housing time, age at turnout and grazing conditions. On the other hand, the second section was focused on coccidiosis and the use of anticoccidials, with questions regarding clinical signs, timing of anticoccidial treatment and reasons for use. A translation of the entire questionnaire (the original of which is in Norwegian) is provided in Online Resource 1. Additional data regarding the breed and numbers of ewes (>1 year on 1 January) reported to the Norwegian Agricultural Authority were collected via NSRS.

### Statistical analysis

Statistical calculations were done in Excel 2013 (Microsoft Inc.) and Stata 14 (Stata Statistical Software: Release 14. College Station, TX: StataCorp LP). For calculations of significance based on means, *t* tests were used. Fisher’s exact test was used for categorical data, while the Pearson correlation coefficient was used for continuous data. *P* < 0.05 was regarded as significant.

## Results

### Questionnaire

**Fig. 1:**
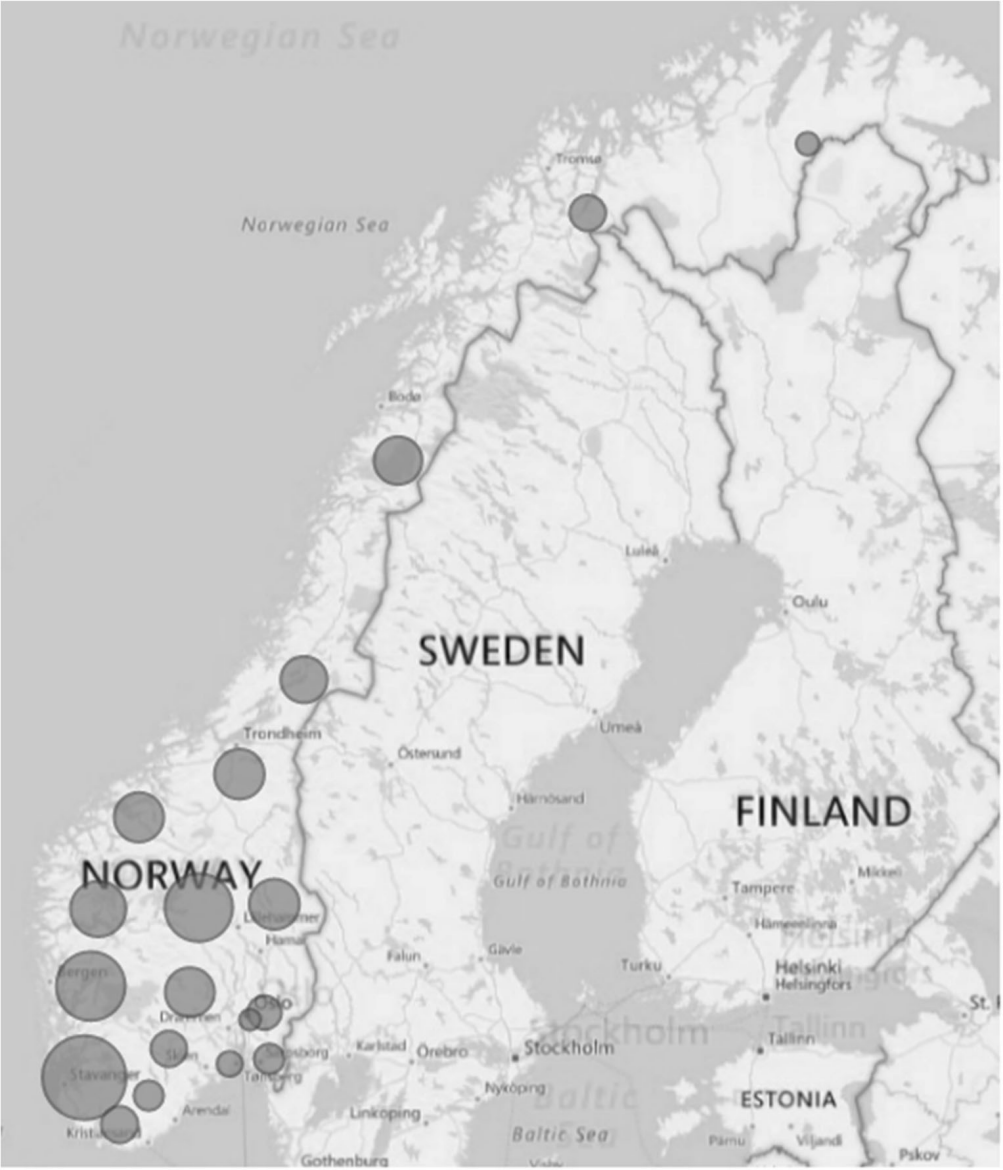
Distribution of sheep farms included in the study, grouped by county. The size of the circle indicates the number of respondents, range 2–228 respondents per county

The final data set consisted of 1096 complete and 119 incomplete questionnaires, of which 6 responded to the advertisement in the Sheep and Goat Farmer’s Journal. This corresponds to a response rate among the NSRS members of 31.3%. When possible, the incomplete questionnaires were included in the analysis. Thus, *n* values vary between calculations. The respondents represent all 19 counties in Norway, with most of the respondents from the west coast and the inland mountain area (Fig. 1). The number of respondents in each county corresponded to the general geographical flock distribution in Norway (Statistics Norway 2016a) and showed a strong correlation (*r* = 0.94).

### Management conditions

**Fig. 2:**
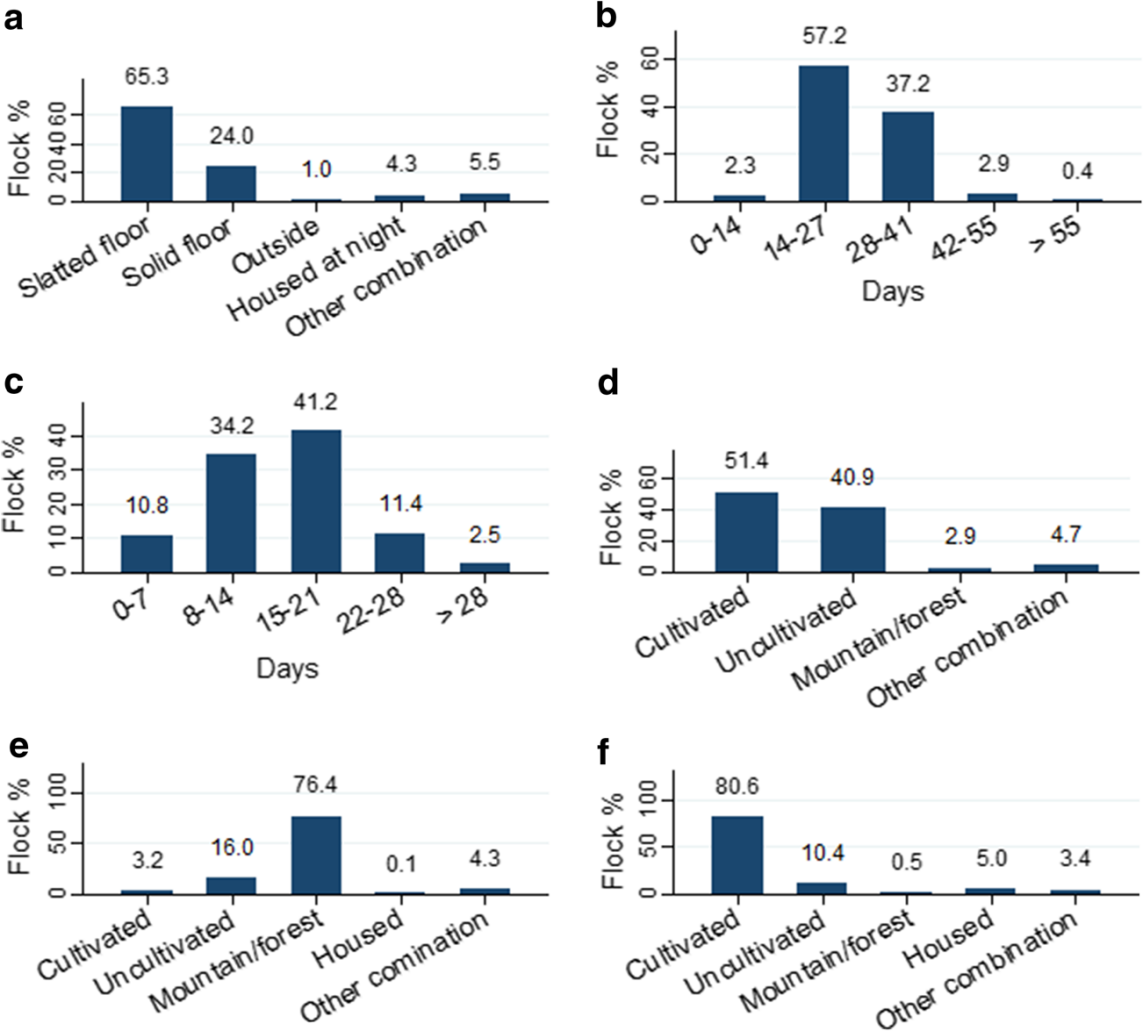
Management of Norwegian sheep farms. **a** Type of housing (*n* = 1152). **b** Duration of lambing period (*n* = 1154). **c** Lamb age at turnout (*n* = 1133). **d** Type of spring pasture (*n* = 1138). **e** Type of summer pasture (*n* = 1138). **f** Type of autumn pasture for lambs (*n* = 1135). Percentages indicated above *bars*

Average flock size (mean ± SEM) was 102.6 ± 2.3 ewes with a range of 1–755. The main sheep breed was Norwegian white sheep (Table 1). Most ewes and lambs were kept on slatted floors (wood, plastic or expanded metal) (65.3%) or on solid floor (straw bedding/wood shavings) (24.0%) (Fig. 2). There was no significant difference (*P* > 0.05) between type of floor and the farmers’ observation of diarrhoea or reduced growth. The lambing period lasted for 14–27 days in 57.2% of the flocks and for 28–41 days in 37.2% of the flocks (Fig. 2). Age at turnout was 0–7 days (10.8%), 8–14 days (34.2%), 15–21 days (41.2%) and 22 days or older (13.9%). Cultivated and uncultivated pasture was used as spring pasture for 51.4 and 40.9% of the flocks, respectively. Lambs were turned out onto pastures that had been used for grazing during the previous spring or autumn in 70.6 and 61.7% of the flocks, respectively, while only 7.9% of the lambs were grazing pastures not used for sheep the previous year. Lambs and ewes were grazing spring pastures for 0–14 days (13.4%), 15–28 days (44.2%) or more than 29 days (42.4%). During summer, 76.4% of the flocks were grazing mountain or forest pastures, and in autumn, 80.6% of the flocks used cultivated pastures for the lambs.

**Table 1:** Total number of ewes and breed distribution per 1.1.2015 in the Norwegian flocks included in the study

Breed	Number of ewes (%)	Number of flocks
Norwegian white sheep (“norsk kvit sau”)	89,224 (74.5)	983
Norwegian white short tail (“kvit spæl”)	12,166 (10.2)	301
Norwegian coloured short tail (“farga spæl”)	2676 (2.2)	157
Old Norwegian short tail (“gammelnorsk spæl”)	2382 (2.0)	125
Norwegian Pelt sheep (“norsk pelssau”)	1845 (1.5)	88
Dala	1455 (1.2)	137
Other breeds	10,083 (8.4)	812

Several flocks had multiple breeds

### Coccidiosis and anticoccidials

Faecal samples for parasitological analysis of gastrointestinal parasites were submitted to diagnostic laboratories from 140 (12.3%) of the flocks during 2014 and 2015. The main reasons for parasitological analyses were (a) surveillance (65.4%), (b) disease (18.4%) and (c) combinations of these (16.2%).

In response to a question on which parasites and parasitic diseases the farmers felt were of relevance in their flocks, 54.0% of farmers selected coccidiosis as being relevant. Other important parasites were nematodes (59.6%), *Fasciola hepatica* (54.0%) and *Ixodes ricinus* (34.3%).

Toltrazuril (Baycox® Sheep vet.) and diclazuril (Vecoxan® vet.) were used in 87.4 and 5.8% of the treated flocks, respectively (Table 2). In 17.3% of the total number of flocks, anticoccidials had never been used. A significant difference in flock size was observed between the farmers that never used anticoccidials (mean flock size 73.3 ± 4.1) compared with farmers that treated with anticoccidials (109.5 ± 2.7) (*P* < 0.05).

**Table 2:** Use of anticoccidial drugs in Norwegian sheep farms included in the study

		Number	Percentage
Treatment	Never	193	17.3
	Not every year	166	14.8
	Annually (last 1–4 years)	179	16.0
	Annually (>4 years)	580	51.9
	Total number of farms	1118	
Purpose	Metaphylactic (previous problems)	551	60.4
	Metaphylactic (no previous problems)	257	28.1
	Therapeutic	84	9.2
	Other	21	2.3
	Total number of farms	913	
Drug	Baycox® Sheep vet. (Bayer Animal Health)	794	87.4
	Vecoxan® vet. (Elanco Animal Health)	53	5.8
	Baycox® Sheep vet. and Vecoxan® vet.	19	2.1
	Sulpha preparations	6	0.7
	Unknown	36	4.0
	Total number of farms	908	
Time	All lambs at turnout	347	38.6
	All lambs 7–10 days after turnout	292	32.4
	Individual lambs with clinical signs	112	12.4
	Other management^a^	149	16.6
	Total number of farms	900	
Frequency	Once per year	746	84.1
	≥Twice	46	5.2
	Selected symptomatic lambs >once	95	10.7
	Total number of farms	887	

^a^Other management includes different treatment times within one flock, e.g. lambs born early were treated a week after turnout, while lambs born later were treated at turnout

In treated flocks, anticoccidials were administered at turnout (38.6%), 1 week after turnout (32.4%), in lambs showing clinical signs (12.4%) or by using a combination of the above (16.6%). According to the farmers, metaphylactic treatment, i.e. treatment in the prepatent period to prevent clinical signs of coccidiosis, was practised in 88.5% of the treated flocks. Of these, one third had no history of clinical outbreaks. The majority of the flocks (84.1%) treated the lambs only once with anticoccidials (Table 2).

**Fig. 3:**
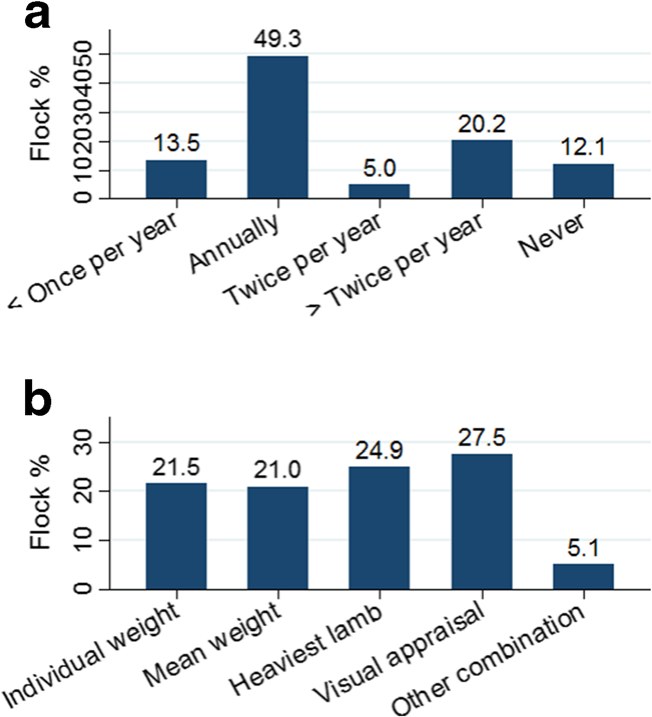
**a** Drench gun calibrations per year (*n* = 901). **b** Methods used for dose estimation (*n* = 903) in Norwegian sheep farms. Percentages indicated above *bars*

Drench gun calibrations were usually performed once each year (49.3%). Dose estimation of anticoccidials was based on the weight of the heaviest lambs and visual appraisal of lamb weight in 24.9 and 27.5%, respectively (Fig. 3). A significant difference in flock size was seen between the farmers using visual appraisal as dose estimation (mean flock size 99.0 ± 4.8), compared with farmers weighing the heaviest animal (129.5 ± 6.7) (*P* < 0.001).

The occurrence of diarrhoea and/or impaired weight gain correlated to the use of anticoccidials is presented in Table 3. Farms with no use of anticoccidials reported significantly less diarrhoea/perineal soiling and a more normal growth rate among lambs during the spring pasture period (Table 3). Additionally, flocks with diarrhoea were significantly larger than flocks without signs of diarrhoea, both during the housing period (mean flock size 115.2 ± 3.8 vs 91.9 ± 2.9) and after turnout (110.7 ± 3.2 vs 91.1 ± 3.5) (*P* < 0.01). Flocks that were described by the farmers as having reduced growth rates were significantly larger than flocks reporting of apparent normal growth rates: during the housing period (mean flock size 116.0 ± 3.8 vs 91.1 ± 3.1) and after turnout (115.5 ± 3.6 vs 88.6 ± 3.3) (*P* < 0.01).

**Table 3:** Presence of diarrhoea and/or reduced weight gain in lambs in Norwegian sheep farms during housing and spring pasture periods, respectively, depending on treatment with anticoccidials or absence of treatment

			Treatment with anticoccidials		No treatment	
			*n*	%	*n*	%
Indoor period	Diarrhoea/perineal soiling	Yes	428	47.2	76	39.8
		No	479	52.8	115	60.2
		Total	907		191	
	Reduced weight gain	Yes	436	51.4	86	46.7
		No	413	48.6	98	53.3
		Total	849		184	
Spring pasture period	Diarrhoea/perineal soiling	Yes	583	63.9	90	47.4**
		No	329	36.1	100	52.6
		Total	912		190	
	Reduced weight gain	Yes	499	58.5	83	45.4*
		No	354	41.5	100	54.6
		Total	853		183	

Statistical (Fisher’s exact) differences between treating/non-treating and the presence or absence of diarrhoea/perineal soiling and reduced weight gain are marked: *P < 0.05, **P < 0.001

In 37.9% of the flocks, the farmers experienced lambs with clinical signs possibly related to coccidiosis after treatment with anticoccidials. These flocks were larger than the flocks not reporting this potential lack of treatment effect (122.9 ± 4.6 vs 101.9 ± 3.4) (*P* < 0.001). However, of these flocks, only 16.7% reported that they submitted faecal samples for parasitological analysis.

## Discussion

In this study, we report the main management practises in Norway regarding coccidiosis in lambs and the use of anticoccidials and link them to potential risk factors for reduced anticoccidial efficacy, i.e. flock size, treatment without a confirmed diagnosis and incorrect dosing due to inaccurate weight estimation and lack of gun calibration.

One important finding of our survey was that more than 80% of the Norwegian sheep flocks were treated for coccidiosis, mainly without a laboratory-based diagnosis or presence of clinical signs. Metaphylactic treatment is recommended for both toltrazuril and diclazuril, based on the mode of action of the drugs and the intention of reducing the massive destruction of the intestinal epithelium, which is particularly severe when the oocysts are excreted (Gregory and Catchpole 1990, 1987). Both drugs act against all intracellular stages in the schizogony and gamogony phases (Haberkorn and Stoltefuss 1987; Harder and Haberkorn 1989; Maes et al. 1989) and have been shown to reduce oocyst excretion efficiently in lambs when administered as metaphylactic treatment (Mundt et al. 2009). During the period 2010–2015, the annual use of Vecoxan® vet. declined from 869 to 379 L, while the annual use of Baycox® Sheep vet. in the same period increased from 2933 to 4985 L (Norwegian Institute of Public Health 2015). The farmers’ and veterinarians’ preference for Baycox® Sheep vet. over Vecoxan® vet. may be linked to the usage of the drug, as Baycox® Sheep vet. Administered at turnout is less time-consuming than the later treatment (Gjerde et al. 2009). In addition, studies have indicated that Baycox® Sheep vet. may have a better effect against ovine coccidiosis than Vecoxan® vet. (Mundt et al. 2009; Gjerde et al. 2009). Sulpha-containing drugs were also used by the farmers although these drugs are not registered for treatment of coccidiosis in Norway.

Almost one third of the farmers treated their flocks, despite clinical coccidiosis not being considered a problem in previous years. Furthermore, the farmers apparently had little knowledge about the actual infection status of their animals, since diagnostic samples were analysed in only around 10% of the farms. These diagnostic samples were analysed for all gastrointestinal parasites, and the percentage of farmers requesting diagnostics particularly for coccidiosis was probably even lower. The potential presence of other infectious agents in young lambs with similar symptoms, such as *Nematodirus battus*, *Cryptosporidium*, *Escherichia coli* and rotavirus (Jackson and Coop 2007; Tzipori et al. 1981; Snodgrass et al. 1976; Munoz et al. 1996) emphasizes the need for a correct diagnosis. In addition, concurrent infections with other microbes can lead to increased severity of the coccidial infection (Catchpole and Harris 1989). Treatment in flocks without previous history of coccidiosis or in the absence of a diagnosis may lead to unnecessary and unsuccessful treatment. Consequently, uncontrolled and extended use of anticoccidials may be a risk factor for the development of ACR in Norwegian sheep farms, as reported for anthelmintics (Barton 1983; Jackson and Coop 2000; Domke et al. 2011).

According to the questionnaire and the widespread use of anticoccidial treatment, most Norwegian farmers are concerned about coccidiosis and consider this disease to be important in their flocks. This concurs with previous results (Gjerde and Helle 1991; Gjerde et al. 2010) in which it was reported that coccidiosis is one of the most important parasitosis affecting Norwegian lambs. Several factors, including stress, poor hygiene during housing, low availability of clean pastures, lack of pasture rotation and the capacity of pathogenic *Eimeria* spp. to overwinter, may be decisive for the widespread clinical problems (Helle 1970; Taylor 2000; Mitchell et al. 2012). For example, poor hygiene at housing, especially related to food and water troughs, has been linked to increased risk of clinical coccidiosis (Taylor 1995; Mitchell et al. 2012). In addition, bad weather during spring may lead to delayed turnout, which can increase the infection pressure during the housing period and affect the farmer’s ability to treat at the optimal time.

Lambs in our study were largely turned out onto permanent pastures used for grazing during the previous spring and/or autumn, thereby increasing the risk of infection (Svensson et al. 1994). In addition, almost 60% of the farmers kept lambs and ewes on spring pastures for more than 3 weeks, which is long enough for the parasite to complete at least one full life cycle. Consequently, the infection pressure increases, explaining why coccidiosis in Norway is usually a problem after turnout. This contrasts with countries such as Iceland, where the lambs are on spring pastures for such a short period that development of immunity is compromised, and therefore, coccidiosis can occur when the lambs are brought back to home pasture in autumn (Skirnisson 2007).

Surprisingly, farmers that treated their flock for coccidiosis reported significantly more diarrhoea and reduced weight gain than untreated flocks during both housing and spring grazing periods. However, this may indicate that farmers were treating their lambs because they were symptomatic. On the other hand, in accordance with the positive correlation between flock size and the use of anticoccidials, farmers with larger flocks reported coccidiosis-related symptoms more frequently. The reason for this observation is unknown, but it may be related to a higher animal density leading to an increased infection pressure, as described for caprine coccidiosis (Ruiz et al. 2006) and nematode infections in sheep (Thamsborg et al. 1998).

Signs related to possible clinical coccidiosis after treatment was observed by almost 40% of the farmers in our study, and the development of ACR could be one explanation. However, as only a few of these farmers had submitted faecal samples for diagnosis, clinical signs could also be related to other infections. Additionally, possible coccidiosis-related symptoms after treatment could be associated with treatment failure due to factors such as poor timing (Enemark et al. 2015) or incorrect storage of the drug (Gradwell 2000). Gjerde and Helle (1986) demonstrated that 20 mg/kg toltrazuril, administered as a single oral dose, is more effective at reducing oocyst numbers than a single oral dose of 10 or 15 mg/kg, so under-dosing is also factor that should be taken into account. Under-dosing is not only a cause of treatment failure but also a well-known risk factor for anthelmintic resistance (Smith et al. 1999; Wolstenholme et al. 2004) and probably also increases the risk of ACR development (Ryley 1980). Inaccurate estimation of animal live weight and lack of drench gun calibration might cause incorrect dosing. Accordingly, farmers are encouraged to calibrate their drench guns at least annually, preferably at each drenching, and to estimate the body weight as accurately as possible, preferably by weighing individual animals. Nevertheless, in about a quarter of the flocks of the present survey, visual appraisal of bodyweight was the basis for calculation of the dose and drench guns used for anticoccidial administration were never calibrated in 12.5% of the flocks. However, this is a marked improvement compared with a previous study by Domke et al. (2011), where almost 80% of respondents used visual appraisal for dose estimation and a quarter never calibrated their drench gun. In the present study, the use of visual appraisal was significantly more common in smaller flocks, suggesting that farmers with larger flocks may be more aware of information concerning correct treatments or consider this to be of greater importance.

High infection pressure, possibly related with herd density, could potentially be a factor promoting treatment failure and/or development of ACR, based on the biological characteristics of the parasite itself. Due to the existence of asexual haploid stages of *Eimeria* spp., resistant mutants will for instance be immediately selected in the presence of a drug at the expense of sensitive forms; this stands in contrast to diploid organisms, where the degree of dominance of resistance genes plays a role (Chapman 1993). In addition, coccidia have an enormous capacity for multiplication in the intestine, and resistant strains may rapidly become the dominant phenotype. On the other hand, there is also a huge untreated refugia consisting of oocysts in the environment. This is one main difference between poultry production and sheep production. Poultry housing is thoroughly cleaned between each batch, which is not the case for lambs due to outdoor grazing.

Apart from herd size, no other significant correlations were detected in the present study between management practises and the possible lack of treatment efficacy. Although other studies have indicated that lambs reared on straw appear to be at particular risk of coccidiosis compared with lambs raised on expanded metal (Berriatua et al. 1994; Taylor et al. 1973), our data demonstrate no apparent correlation between floor type and presence of diarrhoea/perineal soiling and/or reduced weight gain. The reason for this is unknown. However, lambs raised on solid floors with deep litter may be trickle-infected and develop effective immunity without clinical symptoms (Reeg et al. 2005; Catchpole et al. 1993).

Recruitment to the study was mainly from members of NSRS with an email address, and this may be a selection bias, perhaps excluding older farmers or those with more remote locations. However, electronic communication is widespread in Norway. Previous questionnaire-based studies of Norwegian sheep flocks have shown response rates of 12.5–50% (Simensen et al. 2014; Holmøy et al. 2012; Domke et al. 2011), compared with the response rate of 31.3% in our study. The lowest response rate was from a study in which participation was requested by regular mail and the response was via the Internet (Simensen et al. 2014). Studies with higher response rate have used email (Holmøy et al. 2012) and regular mail (Domke et al. 2011) for data collection. Thus, the route of communication seems to have no clear association with response rate.

Among members of the NSRS, the mean flock size is larger, average slaughter weights higher and quality classification of carcasses better than for non-members (National Sheep Recording System 2015). In the present study, the flock size was larger (102.6), than the average flock size in Norway in 2015 (74.1) (Statistics Norway 2016a,b). Although the geographical distribution of the respondents corresponded well with the actual geographical distribution of Norwegian sheep farmers (Statistics Norway 2016a), our analysis may therefore be biased by including farms that were better managed than the average national flock.

## Conclusion

Coccidiosis is considered by farmers to be an important parasitic disease in Norwegian sheep flocks. Accordingly, metaphylactic treatment with anticoccidials seems to be the routine practise in most farms, although it is usually performed without a definitive diagnosis. Farmers also reported lambs with possible coccidiosis-related symptoms after treatment. However, from our data, it cannot be determined whether such potential treatment failure is related to management practises, incorrect administration of the drug, other infections or actual ACR.

## Electronic supplementary material

ESM 1 A translated copy of the Internet-based questionnaire sent out to the farmers. Farmers answering “Sheep are outside all year round” at question 8 received question 9–29, whereas the rest of the farmers received questions 30–60. (PDF 503 kb)
