# Supplementary material for: Treatment against coccidiosis in Norwegian lambs and potential risk factors for development of anticoccidial resistance—a questionnaire-based study
Source: Parasitol Res. 2017 Feb 11;116(4):1237–45. doi: 10.1007/s00436-017-5400-7 (PMC5360830; doi:10.1007/s00436-017-5400-7)
Supplement: Supplementary file 1 — A translated copy of the Internet-based questionnaire sent out to the farmers. Farmers answering “Sheep are outside all year round” at question 8 received question 9–29, whereas the rest of the farmers received questions 30–60. (PDF 503 kb) [file 436_2017_5400_MOESM1_ESM.pdf]

Treatment against coccidosis in Norwegian lambs and potential risk factors for development of anticoccidial resistance – a questionnaire-based study

Parasitology Research

Ane Odden, Heidi L. Enemark, Lucy J. Robertson, Antonio Ruiz, Lisbeth Hektoen, Snorre Stuen

Corresponding author: Ane Odden, Norwegian University of Life Sciences, Faculty of Veterinary Medicine, Department of Production Animal Clinical Sciences, Kyrkjevegen 332/334, N-4325 Sandnes, Norway ane.odden@nmbu.no

Questionnaire regarding coccidiosis

Dear sheep farmer!

This questionnaire is a part of a three-year project to investigate how Norwegian sheep farmers are treating for coccidiosis, and whether the treatment is effective. The reason for the project is that there have been several reports of reduced efficacy of the anticoccidials being used.

Coccidiosis can be suspected when young lambs, often at spring pasture, have diarrhoea and/or reduced weight gain. The cause of coccidiosis is the protozoan parasite, *Eimeria*, which infects the gut epithelium and destroys the cells. Coccidiosis can have a major economic impact, especially if many animals in the flock are affected.

Answers from this questionnaire will give us a better understanding of how we are treating coccidiosis in Norway, and whether the efficacy is as expected. Based on information obtained from this questionnaire, we will invite some farmers to participate in a sampling programme during spring 2016, to assess the efficacy of anticoccidial treatment.

We would like to combine the answers obtained in this questionnaire with data in the Norwegian Sheep Recording System, and we therefore ask your permission to access your data. All data and answers will be handled anonymously.

If you are keeping sheep both housed and outdoors, we would like you to let us know the proportion of your sheep that are housed.

The questionnaire should take 5-10 minutes to complete. All farmers completing the questionnaire will be entered into a draw with the possibility of winning a tablet computer.

Thank you for your participation!

1. Are you a member of the Norwegian sheep recording system?

(Only one answer)

- ❑ Yes
- ❑ No

2. Farm number (10 digits):

_ _

3. Number of winter-fed sheep (ewes >one year at Jan 1st):

_ _

4. Which breeds do you keep? (Specify the percentage of each breed, the total should be 100%)

Norwegian White _________ %

Norwegian White Short Tail _________ %

Norwegian Coloured Short Tail _________ %

Old Norwegian Short Tail _________ %

Old Norwegian Sheep _________ %

Other _________ %

5. Do you have regular health visits from a veterinarian?

(Only one answer)

- ❑ Yes
- ❑ No

6. Do you have organic production?

(Only one answer)

- ❑ Yes
- ❑ No

7. Duration of the lambing period in your flock?

(Only one answer)

- ❑ 0-14 days
- ❑ 14-27 days
- ❑ 28-41 days
- ❑ 42-55 days
- ❑ More than 55 days

8. How are your lambs housed before turnout to pasture?

(Only one answer)

- ❑ Slatted floor: plastic, wood or expanded metal
- ❑ Solid floor with straw or wood shavings
- ❑ Sheep are outside all year round
- ❑ Housed at night, outside at day

Other

_ _

Section A, for farmers answering "Sheep are outside all year round" **at question 8.**

9. Have you submitted faecal samples from your lambs to a veterinary diagnostic laboratory during the last two years?

(Only one answer)

- ❑ Yes
- ❑ No

10. When were the samples taken?

- ❑ Before/at lambing
- ❑ Spring pasture
- ❑ Summer pasture
- ❑ Autumn pasture

11. Why were the samples taken?

- ❑ Clinical disease
- ❑ Reduced growth
- ❑ General surveillance
- ❑ To evaluate the need for treatment in otherwise healthy animals

Other

_ _

12. Which parasites do you consider important in your flock:

(Only one answer per parasite)

	Important	Not important	Uncertain
Ixodes ricinus	❑	❑	❑
Lice	❑	❑	❑

Fasciola hepatica	❑	❑	❑
Dicrocoelium dendritticum	❑	❑	❑
Nematodirus battus	❑	❑	❑
Other nematodes	❑	❑	❑
Tapeworms	❑	❑	❑
Lung worm	❑	❑	❑
Coccidia	❑	❑	❑

13. Have you seen diarrhoea/perineal soiling or reduced weight gain in lambs up to 8 weeks old?

(Only one answer per row)

	In 0-20%	In 20-40%	In 40-60%	In 60-80%	In 80-100%	Not seen
Diarrhoea/ perineal soiling	❑	❑	❑	❑	❑	❑
Reduced weight gain	❑	❑	❑	❑	❑	❑

14. Do you treat against coccidiosis, and for how long have you treated?

(Only one answer)

❑ No, have never treated

❑ Yes, but do not treat every year

❑ Yes, have treated annually for 1-4 years

❑ Yes, have treated annually for 5-9 years

❑ Yes, have treated annually for 10 or more years

15. Which preparation did you use in 2015?

❑ Baycox® Sheep vet

❑ Vecoxan® vet

- ❑ Sulpha-preparations

Other

____

16. For how long have you used the same preparation?

(Only one answer)

- ❑ 1 year
- ❑ 2-5 years
- ❑ 5-10 years
- ❑ More than 10 years

17. How and why do you treat?

- ❑ To prevent clinical coccidiosis: flock has never had clinical coccidiosis previously
- ❑ To prevent clinical coccidiosis; flock has had clinical coccidiosis previously
- ❑ Treatment of clinical coccidiosis

Other

____

18. When do you treat against coccidiosis?

- ❑ All lambs are treated before they are two weeks old
- ❑ All lambs are treated before they are two - four weeks old
- ❑ All lambs are treated when they are older than four weeks
- ❑ Individual lambs are treated if needed, e.g. if they have diarrhoea
- ❑ All lambs are treated when some individual lambs have diarrhoea

- ❑ All lambs are treated regularly, e.g. at every change of pasture
- ❑ Only bottle fed lambs are treated

Other

_ _

19. How many times did you treat the whole flock this spring?

- ❑ Once
- ❑ Twice
- ❑ More than twice
- ❑ Only some individual lambs were treated more than once (not the whole flock)
- ❑ No lambs were treated more than once

20. How do you estimate the dose?

(Only one answer)

- ❑ Individual weighing
- ❑ Weighing of medium-sized/average lamb
- ❑ Weighing of heaviest lamb
- ❑ Visual appraisal

Other

_ _

21. How often do you check that your drench gun gives the correct dose?

(Only one answer)

- ❑ Less than once a year
- ❑ Once a year
- ❑ Twice a year

- ❑ > twice a year
- ❑ Never

22. Have your lambs ever experienced clinical disease/diarrhoea because of what you think is coccidia, even though the lambs have been treated with an anticoccidial?

(Only one answer)

- ❑ Yes
- ❑ No

23. Do you treat adult sheep against coccidiosis?

(Only one answer)

- ❑ Yes
- ❑ No

24. Why do you treat adult sheep against coccidiosis?

- ❑ Diarrhoea during the housed (winter) period
- ❑ Diarrhoea in the spring pasture period
- ❑ Low body condition score

Other

_ _

25. When do you treat adult sheep against coccidosis?

- ❑ At lambing
- ❑ At turnout
- ❑ When the ewes and lambs are sent to mountain/forest pastures

Other

_ _

26. Who are your advisors regarding treatments against parasitic diseases?

(Only one answer per row)

	Not important	Somewhat important	Either or	Important	The most important
Articles in farmers' journals	❑	❑	❑	❑	❑
Internet	❑	❑	❑	❑	❑
Other farmers	❑	❑	❑	❑	❑
Close family	❑	❑	❑	❑	❑
Abattoir adviser	❑	❑	❑	❑	❑
Veterinarians	❑	❑	❑	❑	❑

27. Are you interested in participating in this project? Participation includes faecal sampling of lambs and analysis without cost. The analysis will be especially concerned with coccidia and the efficacy of treatment. If you are interested in participating, we might contact you during winter 2016.

(Only one answer)

❑ Yes

❑ No

28. Thank you for participating! If we need further information, we would like to have your address, email address, telephone number and farm number.

_ _

29. Other comments?

_ _

Section B, for farmers NOT answering “Sheep are outside all year round” **at question 8.**

30. Average lamb age at turnout spring 2015?

(Only one answer)

- ❑ 0-7 days
- ❑ 8-14 days
- ❑ 15-21 days
- ❑ 21-28 days
- ❑ 29 days or older

31. Average spring pasture period for ewes and lambs?

(Only one answer)

- ❑ 0-7 days
- ❑ 8-14 days
- ❑ 15-24 days
- ❑ 22-28 days
- ❑ 29-35 days
- ❑ More than 35 days

32. Which type of pasture was used for spring pasturing?

(Only one answer)

- ❑ Cultivated pastures
- ❑ Uncultivated pastures
- ❑ Direct turn out to mountain/forest pastures

Other

_ _

33. Which type of pasture did the majority of your sheep graze during summer?

(Only one answer)

- ❑ Cultivated pastures
- ❑ Uncultivated pastures
- ❑ Mountain/forest pasture
- ❑ They were housed

Other

_ _

34. What type of pasture/housing did most of your ewes have during autumn?

(Only one answer)

- ❑ Cultivated pastures
- ❑ Uncultivated pastures
- ❑ Mountain/forest pasture
- ❑ Housed

Other

_ _

35. What type of pasture/housing did most of your lambs have during autumn?

(Only one answer)

- ❑ Cultivated pastures
- ❑ Uncultivated pastures
- ❑ Mountain/forest pasture
- ❑ Direct housing after weaning

Other

_ _

36. What type of pasture/housing did the majority of your ewes have during winter?

(Only one answer)

- ❑ Cultivated pastures
- ❑ Uncultivated pastures
- ❑ Housed
- ❑ Housed, with access to an outdoor pen

Other

_ _

37. Did you use spring pastures that had not been used during the previous spring?

(Only one answer)

- ❑ Yes
- ❑ No
- ❑ Both

38. Did you use spring pastures that had not been used during the previous autumn?

(Only one answer)

- ❑ Yes
- ❑ No
- ❑ Both

39. Have you submitted faecal samples for parasitological analysis during the last two years?

(Only one answer)

- ❑ Yes
- ❑ No

40. When were the samples taken?

- ❑ At turnout

- ❑ On spring pasture
- ❑ On summer pasture
- ❑ On autumn pasture
- ❑ At housing

41. Why were the samples submitted?

- ❑ Disease
- ❑ Reduced growth
- ❑ Generel surveillance
- ❑ To assess treatment needs

Other

_ _

Parasites

42. Which parasites do you consider important in your flock:

(Only one answer per parasite)

	Important	Not important	Uncertain
Ixodes ricinus	❑	❑	❑
Lice	❑	❑	❑
Fasciola hepatica	❑	❑	❑
Dicrocoelium dendritticum	❑	❑	❑
Nematodirus battus	❑	❑	❑
Other nematodes	❑	❑	❑
Tapeworms	❑	❑	❑

Lung worm	❑	❑	❑
Coccidia	❑	❑	❑

Cocccidiosis

43. Have you noticed diarrhoea/perineal soiling or reduced weight gain in lambs in the housed period this spring (2015)?

(Only one answer per row)

	In 0-20%	In 20-40%	In 40-60%	In 60-80%	In 80-100%	Not seen
Diarrhoea/ perineal soiling	❑	❑	❑	❑	❑	❑
Reduced weight gain	❑	❑	❑	❑	❑	❑

44. Have you seen diarrhoea/perineal soiling or reduced weight gain in lambs 2 to 6 weeks after turnout this spring (2015)?

(Only one answer per row)

	In 0-20%	In 20-40%	In 40-60%	In 60-80%	In 80-100%	Not seen
Diarrhoea/ perineal soiling	❑	❑	❑	❑	❑	❑
Reduced weight gain	❑	❑	❑	❑	❑	❑

45. Do you treat against coccidiosis, and for how long have you treated?

(Only one answer)

❑ No, have never treated

❑ Yes, but do not treat every year

❑ Yes, have treated annually for 1-4 years

❑ Yes, have treated annually for 5-9 years

❑ Yes, have treated annually for 10 or more years

46. Which preparation did you use in 2015?

- ❑ Baycox® Sheep vet
- ❑ Vecoxan® vet
- ❑ Sulpha-preparations

Other

_ _

47. For how long have you used the same preparation?

(Only one answer)

- ❑ 1 year
- ❑ 2-5 years
- ❑ 5-10 years
- ❑ More than 10 years

48. How and why do you treat?

- ❑ To prevent clinical coccidiosis; flock has never had clinical coccidiosis previously
- ❑ To prevent clinical coccidiosis; flock has had clinical coccidiosis previously
- ❑ Treatment of clinical coccidiosis

Other

_ _

49. When did you treat?

- ❑ All lambs at turnout
- ❑ All lambs 7-10 days after turnout

- ❑ Individual lambs if needed, e.g. diarrhoea
- ❑ All lambs are treated when one lamb shows clinical signs
- ❑ All lambs are treated regularly, e.g. at each pasture change
- ❑ Only bottle fed are treated

Other

_ _

50. How many times did you treat the whole flock this spring?

- ❑ Once
- ❑ Twice
- ❑ More than twice
- ❑ Only some individual lambs were treated more than once
- ❑ No lambs were treated more than once

51. How do you estimate the dose?

(Only one answer)

- ❑ Individual weighings
- ❑ Weighing of medium-sized/average lamb
- ❑ Weighing of heaviest lamb
- ❑ Visual appraisal

Other

_ _

52. How often do you check that your drench gun gives the correct dose of anticoccidial?

(Only one answer)

- ❑ Less than once a year

- ❑ Once a year
- ❑ Twice a year
- ❑ > twice a year
- ❑ Never

53. Have sheep in your flock ever experienced clinical disease/diarrhoea because of what you think is coccidia, even though the lambs have been treated with an anticoccidial?

(Only one answer)

- ❑ Yes
- ❑ No

54. Do you treat adult sheep against coccidiosis?

(Only one answer)

- ❑ Yes
- ❑ No

55. Why do you treat adult sheep against coccidiosis?

- ❑ Diarrhoea in the housed period
- ❑ Diarrhoea in the spring pasture period
- ❑ Low body condition score

Other

_ _

56. When do you treat adult sheep against coccidosis?

- ❑ At lambing
- ❑ At turnout
- ❑ When the ewes and lambs are sent to mountain/forest pastures

Other

_ _

Advisors

57. Who are your advisors regarding treatments against parasitosis?

(Only one answer per row)

	Not important	Somewhat important	Either or	Important	The most important
Articles in farmers' journals	❑	❑	❑	❑	❑
Internet	❑	❑	❑	❑	❑
Other farmers	❑	❑	❑	❑	❑
Close family	❑	❑	❑	❑	❑
Abattoir adviser	❑	❑	❑	❑	❑
Veterinarians	❑	❑	❑	❑	❑

58. Are you interested in participainge in this project during spring 2016; participation involves faecal sampling from lambs?

(Only one answer)

❑ Yes

❑ No

59. Thank you for participating. Please record your name, address, email address, and telephone number.

_ _

60. Do you have any final comments?

_ _
